# Listener’s personality traits predict changes in pupil size during auditory language comprehension

**DOI:** 10.1038/s41598-021-84886-3

**Published:** 2021-03-08

**Authors:** Isabell Hubert Lyall, Juhani Järvikivi

**Affiliations:** Department of Linguistics, University of Alberta, Edmonton, AB T6G 2R3 Canada

**Keywords:** Psychophysics, Psychology and behaviour

## Abstract

Research suggests that listeners’ comprehension of spoken language is concurrently affected by linguistic and non-linguistic factors, including individual difference factors. However, there is no systematic research on whether general personality traits affect language processing. We correlated 88 native English-speaking participants’ Big-5 traits with their pupillary responses to spoken sentences that included grammatical errors, "He frequently have burgers for dinner"; semantic anomalies, "Dogs sometimes chase teas"; and statements incongruent with gender stereotyped expectations, such as "I sometimes buy my bras at Hudson's Bay", spoken by a male speaker. Generalized additive mixed models showed that the listener's Openness, Extraversion, Agreeableness, and Neuroticism traits modulated resource allocation to the three different types of unexpected stimuli. No personality trait affected changes in pupil size across the board: less open participants showed greater pupil dilation when processing sentences with grammatical errors; and more introverted listeners showed greater pupil dilation in response to both semantic anomalies and socio-cultural clashes. Our study is the first one demonstrating that personality traits systematically modulate listeners’ online language processing. Our results suggest that individuals with different personality profiles exhibit different patterns of the allocation of cognitive resources during real-time language comprehension.

## Introduction

Comprehending language is an impressive feat: understand a word in a fifth of a second^1^, and surprisal at an unexpected word in an utterance can be detected in ERP signatures as early as 250 ms after its onset. Given that the duration of a single syllable is roughly 200ms^1,2^, this means that comprehension and surprisal often occur before the offset of the relevant word. In this short amount of time, listeners complete a number of tasks, including identifying the sounds, recognizing the word form, retrieving lexical information associated with it, and integrating it into the prior context^3,4^.

Recent research suggests that in this process, both linguistic and extra-linguistic context—for example visual affordances, world knowledge, and listener and speaker properties—are taken into consideration in parallel with lexical, syntactic, and semantic information^5–7^. For example, violations of what speakers know about the world disrupt the flow of comprehension as rapidly as a semantically ill-fitting word: For example, semantic violations (such as “Dutch trains are sour”) and violations of world knowledge (such as “Dutch trains are white” when they are, in fact, yellow) have been shown to prompt an identical N400 signature in listeners^6^. Language comprehension has further been shown to be influenced by the listener’s working memory capacity (WMC), which modulates initial attention-related processes, and comprehension effort more generally^8^.

Importantly, recent research suggests that language processing is affected by an interplay of individual differences related to both the speaker and the listener, especially the speaker’s perceived or inferred identity. Van Berkum and colleagues^7^ showed an N400 ERP signature, starting around 200-300 ms after the onset of the critical word (“teddy bear”) when it did not fit with the speakers’ perceived identity, for example, when participants heard an adult male say “I cannot sleep without my teddy bear in my arms,” even though the sentence is in itself well-formed. Similar N400 ERPs were also elicited by statements that did not align with the speaker’s perceived gender or socio-economic status. They also showed that such statements, inconsistent with inferences about the speaker, elicited the same type of response as semantic violations, such as “You wash your hands with horse and water,” as opposed to “You wash your hands with soap and water.” Importantly, Van den Brink and colleagues^9^ showed that this effect was modulated by the listeners’ ability to empathize: high empathizers showed a larger N400 effect than low empathizers, and the size of the N400 amplitude was significantly correlated with the participants’ Empathy Quotient Questionnaire score. In the same vein, Grant and colleagues^10^ showed that semantically incongruous words in statements involving gender stereotyped role names elicited a larger N400 when the sentences were spoken by a stereotype incongruent voice (male vs. female). Moreover, the authors found that participants that scored higher on Ambivalent Sexism Inventory were less likely to show adaptation to these incongruencies in the course of the experiment.

Using similar materials to Van den Brink and colleagues^9^, Hubert and Järvikivi^11^ found that listeners who scored higher on the Disgust scale (*DS-R*^12,13^) experienced significantly larger pupil dilation than listeners lower on the scale when they came across a statement which clashed with the speaker’s perceived stereotypical gender identity, such as “I always buy my ties at Hudson’s Bay” spoken by a female speaker. Disgust sensitivity, the emotional signature of the Behavioural Immune System, protects an individual from pathogen contamination by behavioural means^14^ and affects aspects of general cognition; for example, individuals more sensitive to disgust tend to engage more in outgroup stigmatization and to oppose open immigration^14–16^. In line with this, disgust sensitivity has been shown to correlate with individual’s political and moral views^14^, which, in turn, have been shown to affect the comprehension of statements involving moral conjectures^17^. Importantly, disgust sensitivity is related to more general personality traits, correlating with Extraversion and Openness, as well as the Honesty-Humility factor in the HEXACO measure^18^. Even though the evidence is still scarce, taken together, these results point to influences of (at least some aspects of) personality on listeners’ language processing performance. However, there is to date no systematic study researching how personality correlates with listeners’ cognitive resource allocation during real-time language comprehension. The present study will begin to bridge this gap between more general personality traits and language comprehension by investigating the effect of listeners’ Big 5 traits on the processing of spoken statements that violate grammatical, semantic, and pragmatic expectations.

An individual’s personality traits influence many different aspects of a person’s life, including, but not limited to, academic motivation, success, and the choice of learning style^19^, work performance^20,21^, the choice of romantic partners and friends^22^, and social media use^23^. At least a subset of some personality traits is believed to have a physiological basis, such as the Introversion/Extraversion dimension relating to optimal arousal levels^24,25^, and to different activity levels in certain brain regions^24^.

In the linguistic realm, personality affects patterns of language use, for example narrative styles and lexical choice^26^, social media language use^27^, multiple aspects of second language learning^28^, reading fluency^29^, and comprehension of irony^30^. Experimental results suggest that the author’s Extraversion can be deduced accurately based only on written text output^31,32^.

In a recent study, Boland and Queen^33^ had participants read (supposed) email replies to an ad looking for a new housemate, rating how much they would want the author of a particular reply to be their housemate. Results indicate that the reader’s personality traits interacted with the presence of two different types of errors, “grammos” (such as *to* for *too*) and “typos” (such as *teh* for *the*), in an email to affect the reader’s ratings of the prospective housemate: Less agreeable readers judged grammos more harshly, whereas the same was true for less open readers in response to typos.

In this study, we investigated auditory language comprehension in adults, correlating their pupil sizes in response to sentences (anomalous vs. a non-anomalous baseline) with their Big Five personality traits. Participants were listening to spoken sentences with three types of violations compared to their non-anomalous counterparts: Morpho-syntactic errors violating the agreement rules of English (“He frequently *have* burgers for dinner after work”); semantic anomalies (“Dogs sometimes chase *teas* on the road for fun”); and social-cultural clashes that violated assumptions related to stereotyped gender roles, as inferred from the speaker’s voice (“I usually wear *lip gloss* to work and at home,” spoken by a male voice). In what follows, we will refer to all these incongruencies as violations, and their congruous counterparts as baseline, while at the same time acknowledging the difference in kind between these three types (from normative, grammatical rules to stereotyped inferences). As we were specifically interested in how personality would affect the processing of statements that violate stereotypical gender-based expectations, all experimental stimuli were spoken by both a male and a female speaker.

The size of the human pupil is considered an indicator of autonomic nervous system activity^34,35^ that is responsive to cognitive effort, mental workload, attention, arousal, and affective processing^34,36,37^. In language science, pupillometry has been shown to respond to the intelligibility of speech^38,39^, listening effort^40,41^, sentence complexity^37,42^, ambiguity^43,44^, and semantic anomalies^45^. An important advantage of pupillometry over paradigms that require overt action or input is that language comprehension processes can be analyzed in the absence of a task which might otherwise directly draw attention to the phenomenon under investigation. Pupillometry can also reliably detect individual difference effects^11^. Beyond linguistic stimuli, Gingras and colleagues^34^ showed that pupil size is correlated with the arousal and tension ratings of musical excerpts. Considering that this study also found gender differences, and differences based on how big of a role music played in the listener’s life, results suggest that both the quality of the stimulus and the background and experiences of the listener affect pupil dilation.

Of importance for our study is *vocal gender*, that is, the gender of a speaker as inferred solely from their voice. Voices interpreted as male generally have lower formant frequencies, a lower fundamental frequency, and greater resonance^46^. Expectations around how a speaker of a certain gender ought to sound have been found to even affect lower-level comprehension processes, such as the perception of speech: For example, expectations regarding how a speaker of a certain gender should sound can affect the perception of the physical speech signal, resulting in different perception of phoneme contrasts^46^. In a recent study, vocal gender affected the comprehension of passages either congruent or incongruent with stereotypically male or female occupations, modified by how sexist each individual listener was^10^. Vocal gender is thus a good testing ground for research at the intersection of the listener’s personality traits and stereotypical inferencing about the speaker, as we will demonstrate below.

Based on previous research, we expected a significant increase in relative pupil size for all types of violation—morpho-syntactic errors, semantic anomalies, and socio-cultural violations—as compared to baseline. Crucially, as recent studies suggest an influence of empathy, disgust sensitivity, and Neuroticism, we also expected those changes in relative pupil size to be modulated by an individual’s personality traits, especially in the case of socio-cultural violations. Given that listeners’ moral views^17^ and disgust sensitivity^11,47^ modulate the ease with which they process statements that deviate from personal preferences or stereo-typical state of affairs, we might expect individuals high on the Neuroticism scale, or low on the Openness scale, to experience larger resource allocation (expressed via an increase in pupil size) when coming across a socio-cultural violation.

## Methods

### Participants

A total of 107 participants completed the experiment for this study. All methods were carried out in accordance with relevant guidelines and regulations. The research and data collection were collected in a manner consistent with ethical standards for the treatment of human participants, as outlined in the *Tri-Council Policy Statement: Ethical Conduct for Research Involving Humans* (TCPS 2 2018), and reviewed and approved by the Research Ethics Board 2 at the University of Alberta (reference number Pro00077213). Informed written consent was obtained from all participants. The two under-18 participants were students at the University of Alberta who consented in writing to their participation for course credit and voluntarily signed up to participate in the present study. Thus, we are in compliance with the Nature Research Journals’ editorial policies regarding articles reporting on studies involving human participants. Further, Chapter 3 of the Tri-Council Policy Statement: Ethical Conduct for Research Involving Humans (TCPS 2), the federal guiding policy for ethical treatment of human participants in Canada, specifies that: Rather than an age-based approach to consent, TCPS 2 (2018) advocates an approach based on decision-making capacity as long as it does not conflict with any laws governing research participation.17-year-old university students, who have been accepted to their undergraduate program, make decisions about their course work, and are able to sign up for experiments via the participant pool, clearly show decision-making capacity as outlined in TCPS 2 (2018) and by the Research Ethics Office at the University of Alberta. While non-native speakers of English were invited to participate in the experiment, their data (n = 11) was removed prior to analysis as a non-native command of English may interfere with language comprehension—for example, it may affect the perceived emotionality of an utterance^48,49^. Additionally, the data from participants whose comprehension question accuracy was below 80% were removed (n = 8), as in those cases attention to or comprehension of the stimuli could not be guaranteed.

The analyses in this paper are thus based on the data obtained from 88 native speakers of English (male/female = 17/71 (19%/81%); age min/max = 17/83, mean [SD] = 23.1 [11.6] years). 66 of those participants (male/female = 12/54) were recruited from the University of Alberta undergraduate linguistics students pool, and received course credit for their participation. An additional 22 participants (male/female = 5/17) were recruited from the general population, not limited to the University of Alberta campus or to an academic background, and received a small monetary compensation for their participation.

### Materials

240 sentence stimuli were created, distributed among the following conditions:

- **Morpho-syntactic errors:** 56 stimuli in total, half of which violated subject-verb agreement, such as “She usually *drive* her car slowly in the snow” instead of “She usually *drives* her car slowly in the snow”^50,51^;
- **Semantic anomalies:** 32 stimuli in total, half of which contained a semantic mismatch between the verb and the object, such as “People often read *heads* for pleasure at night” instead of People often read *books* for pleasure at night”^50,51^;
- **Socio-cultural violations:** 120 stimuli in total, half of which contained a violation of expectations as per common social/gender stereotypes, such as “I sometimes buy my *bras* at Hudson’s Bay,” spoken by a male speaker, as opposed to, for example, “I sometimes buy my *ties* at Hudson’s Bay”^7,9^; the violation thus depends on the gender of the speaker as inferred from their voice; and
- **Unrelated filler sentences:** 32 non-anomalous filler sentences, such as “Chickens normally live in a coop.”

While the focus of this paper is on how the listeners’ personality traits interact with the comprehension of socio-cultural violations, morpho-syntactic errors and semantic anomalies were included as well to compare these pragmatic deviations, which violate common expectations given the context, to more language-internal, structural and semantic-level violations. Both morpho-syntactic errors and semantic anomalies have been shown to result in processing delays for reading and listening times^50–52^, as standardly exemplified in augmented P600 and N400 ERP amplitudes, respectively^1,52,53^, but also in larger pupil dilation^42,45,54^.

All sentences followed the same syntactic pattern to ensure comparability across regions. For item recording, items were presented to one male and one female native speaker of Western Canadian English in random order and recorded in a sound-treated booth using a *MR-2000S* studio recorder with a *Countryman E6* earset microphone (both Korg Inc., Tokyo, Japan) with a sampling rate of 44,100 Hz, and saved as .wav files. Sentences where the waveform was clipped, or in which the prosody sounded noticeably different from those of other items, were re-recorded with the speaker. Experimental sentences were then distributed across four lists, which each list containing 60 sentences of the socio-cultural violation type; 28 sentences of the morpho-syntactic error type; and 16 sentences of the semantic anomaly type. Lists were counterbalanced for error condition (non-anomalous baseline vs. anomalous) and speaker gender (male vs. female), such that each individual participant listened to each statement only once (in one condition, spoken by one speaker).

Each list further included the same 32 unrelated filler sentences (16 spoken by a male speaker, 16 spoken by a female speaker), thus resulting in 136 total sentences (i.e. trials) per list. Each participant was assigned randomly to one list and, accordingly, heard each sentence only once, in just one condition and spoken by one speaker.

Additionally, all items were rated for acceptability in a separate Likert-style ratings experiment, by a separate set of participants (99 native speakers of English recruited from the pool of undergraduate linguistics students at the University of Alberta; male/female = 59/40 (60%/40%); age min/max = 17/31; mean = 20.4 years). While this is a separate experiment that we are not reporting on in detail here, this off-line ratings study also found effects of the listener’s personality on item ratings^47^. The mean per-item ratings resulting from this ratings experiment were fed into the statistical models reported below as a numerical predictor. Numerical ratings were preferred over a simple binary error distinction, as they provide a more fine-grained assessment recognizing the inherent gradient nature in the perception of semantic anomalies and socio-cultural violations.

In the main experiment, a comprehension question was presented to the participant after approximately 30% of items (i.e. each participant was presented with a question after 38 to 41 items total). Questions were simple yes/no questions in line with well-established world knowledge, such as “Do giraffes have long necks?” after the unrelated filler item “Giraffes always have very long necks,” to check for both attention to the experiment, and comprehension of the auditory stimuli that were presented^50,55^.

### Experimental procedure

After introducing the participants to the experimental setup, they were seated in an adjustable chair in a dimly lit sound-treated booth at the Centre for Comparative Psycholinguistics at the University of Alberta. Lighting levels were kept constant throughout the experiment, and for all subjects. While the participants’ movements were not restricted, they were asked to place their head on a chinrest to provide additional stability and a constant screen-to-eye distance. Participants were then instructed to follow the instructions on the screen to calibrate the eye-tracker, and to complete the experiment. During the experiment, stimuli were presented via studio loudspeakers at a comfortable level, and the pupil size of the participant’s right eye was recorded at 250 Hz using an *EyeLink 1000* system (SR Research Ltd., Mississauga, Canada) on a desktop PC.

Each trial began with a one-point drift correct, and, immediately after, the display of a fixation cross at the centre of the screen. Pupil size was recorded from the start of the fixation cross onwards. 2,000 ms later, the audio stimulus began to play, and pupil size was recorded until 500 ms after audio offset. After approximately 30% of trials, participants were presented with a simple comprehension question. After an inter-stimulus interval of 3,000 ms, to allow pupil dilation to return to baseline, the next trial began. Participants were given a short break approximately every thirty-five trials; the length of these longer breaks was up to the participant. The main experiment took between 20 and 30 min to complete. After completing the main experiment, participants then moved on to the post-tests described below.

### Post-tests

Participants completed two post-test questionnaires after the main experiment session, so as not to prime them towards the purpose of the study. Data on the participants’ language background was collected via a pen-and-paper language background questionnaire, and personality traits were assessed using the Big Five personality inventory^56^, coded in *E-Prime 2* ^57^. An overview of the traits assessed with the Big Five test, with examples of associated attributes, is provided in Table 1. The Big Five inventory was chosen for its frequent and continued use in psychological research, and/or because it assesses various aspects of an individual’s personality rather than just providing one overall score.

**Table 1:** An overview of the Big Five traits^56^ used to assess the participants' personality, and traits associated with high scores in the respective scale.

Trait	Attributes associated with high score
Openness	Curious, inventive, creative, unconventional
Conscientiousness	Organized, efficient, responsible, dependable
Extraversion	Sociable, outgoing, energetic, talkative
Agreeableness	Cooperative, friendly, sympathetic, compassionate
Neuroticism	Sensitive, nervous, irritable

As well, correlations between the five personality traits were assessed to inform predictor selection during model fitting. The highest correlation observed was between Openness and Extraversion, at r = 0.30 (p = 0.005), with two additional correlations being significant, namely between Agreeableness and Conscientiousness (r = 0.23, p = 0.03) and Agreeableness and Extraversion (r = 0.24, p = 0.03; for an overview, see Table 2).

**Table 2:** Correlation table for the Big Five personality traits, as observed in the participant sample. Significance levels are indicated with asterisks.

	Openness	Conscientiousness	Extraversion	Agreeableness	Neuroticism
Openness	1.00	0.18	0.30**	0.02	-0.13
Conscientiousness		1.00	0.08	0.23*	0.01
Extraversion			1.00	0.24*	-0.16
Agreeableness				1.00	-0.18
Neuroticism					1.00

It should be noted that, while we attempted to expand our college sample by recruiting external participants as well, our participant sample still skews young; further information on the Big Five trait distributions across genders and recruitment strategies is presented in the Supplementary Materials. In this context, it is important to note that research has generally found older individuals to be higher in Conscientiousness and Agreeableness and lower in Neuroticism, whereas Extraversion and Openness seem to be relatively consistent across the lifespan^58^. However, results are not entirely clear-cut; some research has found older individuals to be less extraverted and open, and more agreeable and conscientious, than younger individuals across different cultures^59^. In either case, future research may benefit from a wider age range in the participant sample, such that relationships between age and personality traits may be assessed statistically.

## Results

Data from eight participants was removed as their comprehension question accuracy rates were below 80% (min = 75%, max = 100%, mean = 93.6%), and comprehension of or attention to the experiment could hence not be guaranteed.

### Data pre-processing

The raw pupillometry data was pre-processed in *R*^60^ and *RStudio* (Version 1.3.959, *Middlemist Red*^61^) with one pupil size sample being one data point. Blinks and the adjacent 20 data points (10 to the left, 10 to the right) were removed using a combination of Jacolien van Rij’s *removeBlinks()* function and a cross-check using velocity thresholds. Missing data was not interpolated, since mixed-effect models, such as the kind used in our analyses (see below), can handle missing data natively^43^. Timestamps were centred around the onset of the target word. Baseline pupil sizes were calculated per participant per trial, with each pupil size sample thus representing the difference between the observed pupil size and the participant-by-trial baseline. Data points further than 2.5 SD’s from the respective baseline (3% of total data points) were removed.

### Model fitting

All results reported below were obtained through generalized additive mixed effects modelling (GAMM) using the *mgcv* (Version 1.8-28^62^) and *itsadug* (Version 2.3^63^) packages, with relative pupil size as the dependent variable. Visualizations of raw data were produced using *ggplot2* (Version 3.2.1^64^) and *ggpubr* (Version 0.3.0^65^). All models included a random smooth for participant by time, and a random intercept by item to account for individual differences within the stimuli, and for random variance between participants beyond the factors of interest. GAMM modelling is well suited to time-series data, such as pupillometry data, as it is able to capture non-linear interactions between continuous predictors without losing information in time-binning^43^.

Data in the time window from 200 ms before the onset of the target word to 2000 ms after was analyzed. All models were fitted using a forwards step-wise selection procedure, where the inclusion of variables was evaluated using a combination of a χ2 test of REML scores via the *compareML*() function, visual inspection, and the estimated p-value of the smooth parameter via the *report_stats*() function^43^. Due to the inherent gradient nature of semantic anomalies and socio-cultural violations, all models included average item ratings, obtained from a separate set of raters^47^, as a predictor.

Of special interest were the three-way interactions between a personality trait, time since target word onset, and average item rating. Separate models were fitted for each individual difference variable, so as to not over-complicate each GAMM; however, each individual predictor that was found to be significant was then fed into a GAMM together with each of the other significant predictors, to test if the effects remained. So, for example, if Openness and Extraversion surfaced as significant predictors in separate models, an additional GAMM was fitted with both Openness and Extraversion as predictors, to check that the effects did not cancel each other out. All effects reported below remained in tests of this kind.

### Morpho-syntactic errors

**Figure 1:**
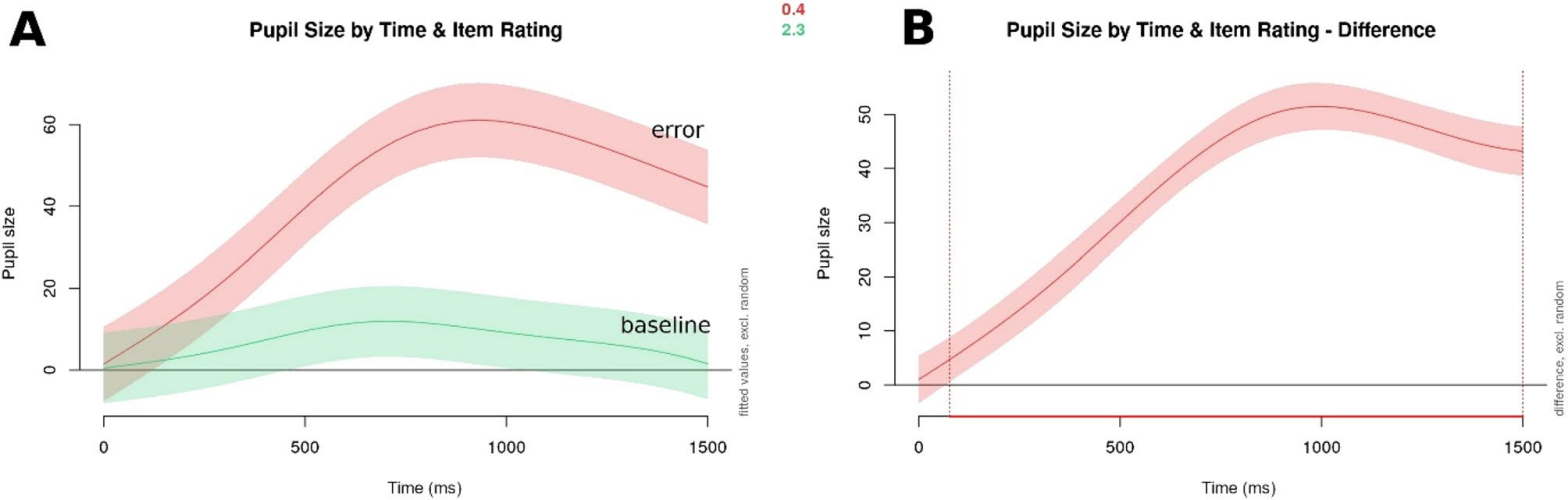
Visualizations of pupil size by item rating over time (in ms since target word onset). Panel A shows baseline (green/lower curve) vs. morpho-syntactic error condition (red/upper curve), and Panel B visualizes the difference between the two, with the red line on the x-axis (below the plot) highlighting the time frame where the difference in pupil size between the baseline and error conditions is significantly different. The shaded areas denote the 95% confidence interval.

A significant interaction was found between item rating (error condition) and time since target word onset: participants showed increased pupil dilation when a statement contained an error than when it did not (see the model output in Table 3 for details, and Fig. 1 for visualizations; χ^2^ (5) = 438.384, p < 0.001 as compared to a basic model containing only time as a predictor variable, with the same random structure).

**Table 3:** Summary output for the basic GAMM modelling participants' pupil sizes in response to morpho-syntactic errors.

Term	edf	ref.df	F-statistic	p value	p level
s (time)	6.292	6.989	19.491	< 0.001	***
s (rating)	8.818	8.986	147.671	< 0.001	***
ti (time, rating)	13.596	15.217	42.441	< 0.001	***
s (participant, time)	578.073	792	34.887	0.039	*
s (item.id)	100.79	102	84.2	< 0.001	***

**Figure 2:**
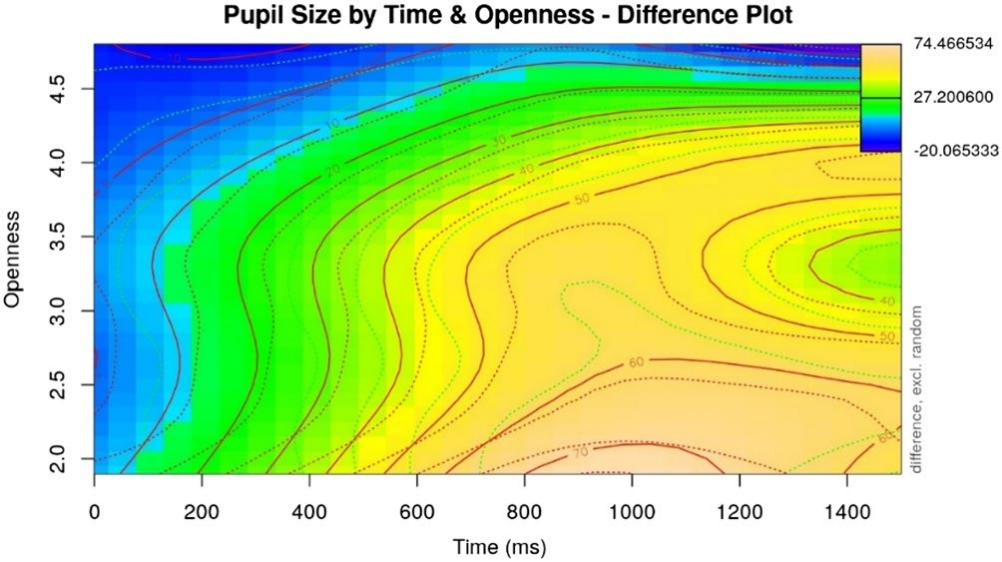
Visualization of the effects of item rating and the listener’s Openness on pupil size over time in response to morpho-syntactic errors. Plot was generated using the itsadug (Version 2.3^63^) package in R.

In GAMMs assessing each Big Five trait as a model predictor, no significant effects were found for Conscientiousness, Extraversion, Agreeableness, and Neuroticism. However, for the participant’s Openness, a significant effect emerged, by which less open listeners showed a larger pupil dilation over time compared to more open listeners (see Table 4 for the model summary, and Fig. 2 for the visualization; χ^2^ (12) = 151.197, p < 0.001 as compared to a model without the Openness variable).

**Table 4:** Summary output for the GAMM modelling participants' pupil sizes in response to morpho-syntactic errors and with their Openness score as a predictor.

Term	edf	ref.df	F-statistic	p value	p level
s (time)	6.283	6.982	18.92	< 0.001	***
s (rating)	8.803	8.984	115.444	< 0.001	***
ti (time, rating)	13.856	15.335	34.6	< 0.001	***
s (open)	1.009	1.009	2.415	0.119	*ns*
ti (open, time)	2.258	2.376	2.268	0.164	*ns*
ti (open, rating)	15.266	15.912	36.438	< 0.001	***
ti (open, time, rating)	49.786	57.842	6.521	< 0.001	***
s (participant, time)	574.549	792	35.511	0.135	*ns*
s (item.id)	100.806	102	84.974	< 0.001	***

Like all surface plots in this paper, Fig. 2 visualizes a three-way interaction between time after the onset of the target word (on the x-axis), one of the listener’s Big Five traits (on the y-axis; here, Openness), and difference in item rating. The participant’s pupil size is represented as a colour scale on the z-axis, and differences in colour visualize the difference in pupil size between the error condition, and the non-anomalous baseline. Here, the colour scale indicates the difference in pupil size when a listener encounters a morpho-syntactic error as compared to the correct baseline. A blue colour indicates a small (or even negative) change in pupil size when listening to a morpho-syntactic error as compared to baseline, whereas a yellow or orange colour indicates a larger dilation; also note the demarcation lines indicating value boundaries. We thus see the three-way interaction of interest visualized as follows in Fig. 2: For example, at 600 ms from the target word onset (x-axis), we see that less open participants show the largest dilation, indicated by the darkest orange colour. As we go up on the y-axis, the colour turns lighter yellow, green, and finally deep blue, indicating that, as Openness scores increase, the difference in pupil size between encountering the baseline and the unexpected words grows smaller. This effect becomes more pronounced moving to the right (in time, on the x-axis), and smaller the closer the time of the pupil sample is to the onset of the target word (that is, moving left on the x-axis). We also see that the lower the listener’s Openness score, the earlier we can see an effect (moving up on the y-axis; cf. the yellow and light orange colours along the bottom and right edges of the plot).

### Semantic anomalies

**Figure 3:**
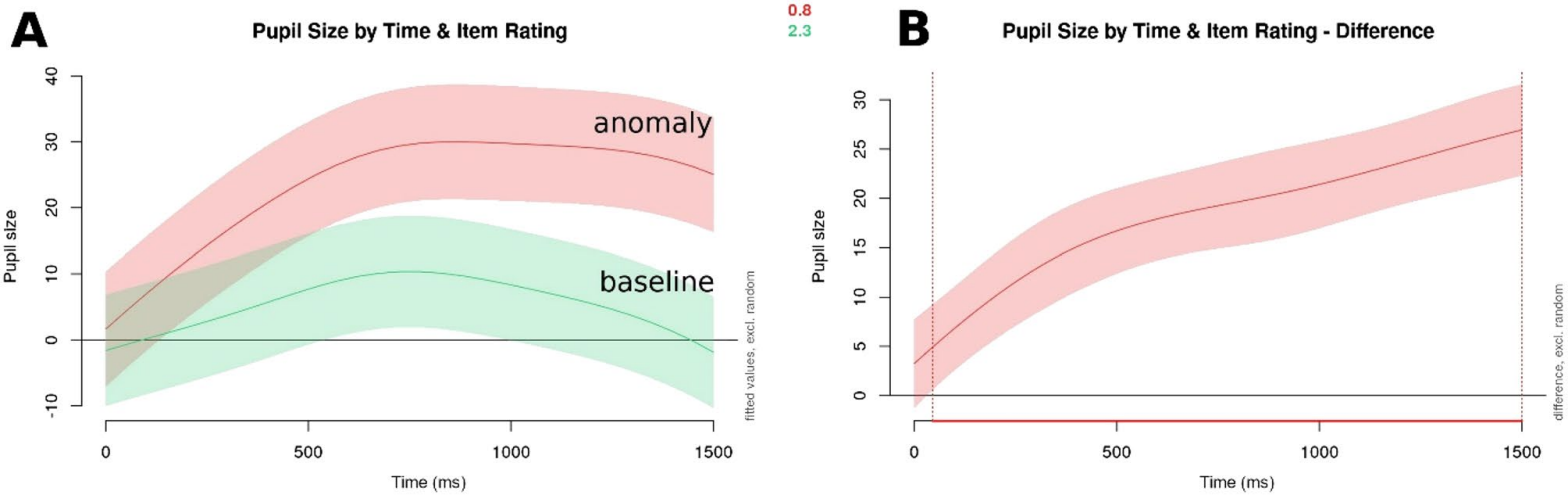
Visualizations of pupil size by item rating over time (in ms since target word onset). Panel A shows baseline (green/lower curve) vs. semantic anomaly condition (red/upper curve), and Panel B visualizes the difference between the two, with the red line on the x-axis (below the plot) highlighting the time frame where the difference in pupil size between the baseline and error conditions is significantly different. The shaded areas denote a 95% confidence interval.

Similarly to the morpho-syntactic condition, an effect of item rating and time since the onset of the target word was found for semantic anomalies as well (see Table 5 for the model summary, and Fig. 3; χ^2^ (5) = 334.165, p < 0.001 as compared to a basic model containing only time as a predictor variable, with the same random structure).

**Table 5:** Summary output for the basic GAMM modelling participants' pupil sizes in response to semantic anomalies.

Term	edf	ref.df	F-statistic	p value	p level
s (time)	5.707	6.434	18.499	< 0.001	***
s (rating)	8.855	8.988	143.076	< 0.001	***
ti (time, rating)	12.771	14.742	17.751	< 0.001	***
s (participant, time)	550.351	792	30.357	0.029	*
s (item.id)	100.696	102	88.441	< 0.001	***

**Figure 4:**
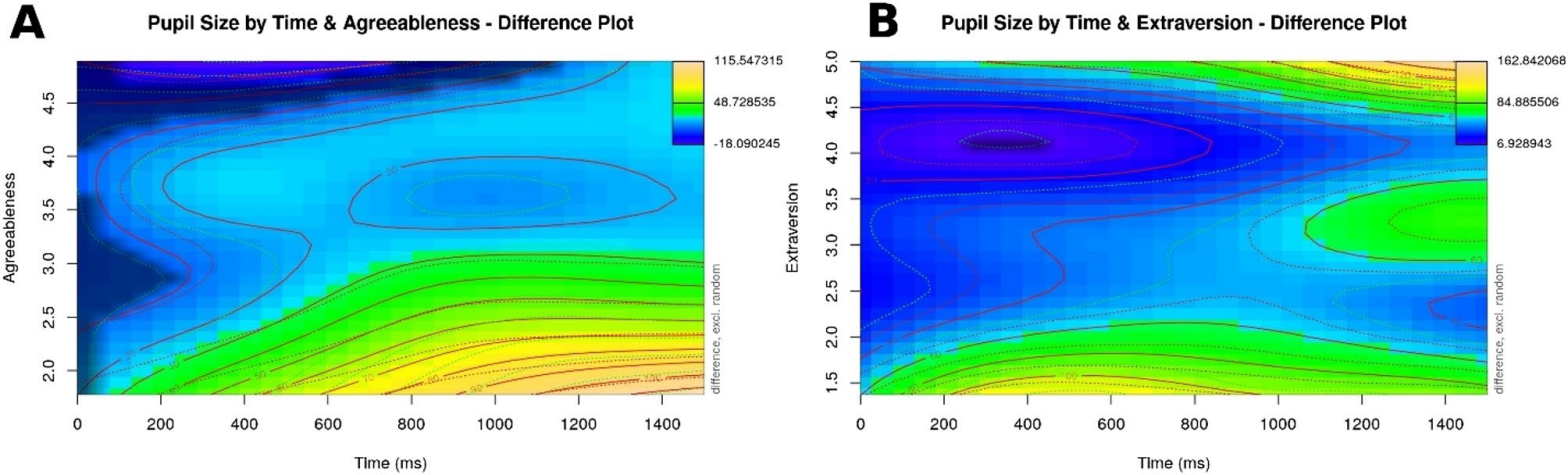
Visualization of the effects of item rating and the listener’s Agreeableness (Panel A) and Extraversion (Panel B) on pupil size over time in response to semantic anomalies. Plots were generated using the itsadug (Version 2.3^63^) package in R.

Further, we found an interaction between Agreeableness, time, and item rating: Less agreeable listeners showed a larger increase in pupil size than their more agreeable peers over time for semantically anomalous statements as compared to baseline (see the model summary in Table 6; χ^2^ (12) = 242.280, p < 0.001 as compared to a model without the Agreeableness variable). As Panel A in Fig. 4 shows, this effect appears around 200 ms from target word onset, growing gradually stronger over time for less Agreeable listeners.

**Table 6:** Summary output for the GAMM modelling participants' pupil sizes in response to semantic anomalies and with their Agreeableness score as a predictor.

Term	edf	ref.df	Statistic	*p* value	*p* level
s (time)	5.738	6.466	18.273	0	***
s (rating)	8.852	8.988	117.521	0	***
ti (time, rating)	12.373	14.382	15.067	0	***
s (agr)	1.006	1.007	3.158	0.075	ns
ti (agr, time)	1.334	1.396	2.031	0.133	ns
ti (agr, rating)	15.346	15.927	52.465	0	***
ti (agr, time, rating)	44.678	53.466	8.701	0	***
s (participant, time)	548.065	792	26.848	0.098	ns
s (item.id)	100.7	102	88.459	0	***

A second trait found to be significant in an interaction with time and item rating was the listener’s Extraversion (see the model summary in Table 7; χ^2^ (12) = 310.396, p < 0.001 as compared to a model without the Extraversion variable). This effect is less straightforward than the effect of Agreeableness above: As Panel B in Fig. 4 shows, less extraverted participants showed a larger increase in pupil size relatively soon (around 200 ms) after the onset of the semantic anomaly; highly extraverted listeners on the other hand experienced a late dilation, around 1,000 ms after target word onset.

**Table 7:** Summary output for the GAMM modelling participants' pupil sizes in response to semantic anomalies and with their Extraversion score as a predictor.

Term	edf	ref.df	Statistic	*p*value	*p* level
s (time)	5.662	6.382	18.079	< 0.001	***
s (rating)	8.86	8.989	158.415	< 0.001	***
ti (time, rating)	12.878	14.732	16.913	< 0.001	***
s (extr)	1.003	1.003	0.016	0.9	ns
ti (extr, time)	1.04	1.048	0.002	0.966	ns
ti (extr, rating)	15.605	15.97	64.532	< 0.001	***
ti (extr, time, rating)	53.629	60.095	11.14	< 0.001	***
s (participant, time)	551.343	792	28.406	0.027	*
s (item.id)	100.695	102	88.759	< 0.001	***

Since two Big Five traits were found to be significant predictors of pupil size in response to semantic anomalies, and since those two traits were found to be weakly correlated (see Table 2 for details), an additional GAMM was fitted (see the model summary in Table 8) that combined the traits of Extraversion and Agreeableness. In this test, both effects remained (comparison to Agreeableness model: c2 (12) = 378.985, p < 0.001; comparison to Extraversion model: c2 (12) = 310.869, p < 0.001, confirming that both Extraversion and Agreeableness contribute significantly to model fit.

**Table 8:** Summary output for the combined GAMM modelling participants' pupil sizes in response to semantic anomalies and with their Agreeableness and Extraversion scores as predictors.

Term	edf	ref.df	F-statistic	p value	p level
s (time)	5.707	6.429	18.158	< 0.001	***
s (rating)	8.852	8.988	138.679	< 0.001	***
ti (time, rating)	12.421	14.381	15.091	< 0.001	***
s (extr)	1.005	1.005	0.434	0.511	ns
ti (extr, time)	1.08	1.096	0.268	0.623	ns
ti (extr, rating)	15.62	15.971	76.906	< 0.001	***
ti (extr, time, rating)	54.496	60.541	13.251	< 0.001	***
s (agr)	1.004	1.004	3.483	0.062	ns
ti (agr, time)	1.104	1.124	2.497	0.106	ns
ti (agr, rating)	15.429	15.942	64.938	< 0.001	***
ti (agr, time, rating)	46.001	54.129	11.134	< 0.001	***
s (participant, time)	549.078	789	25.277	< 0.001	***
s (item.id)	100.709	102	89.043	< 0.001	***

### Socio-cultural violations

**Figure 5:**
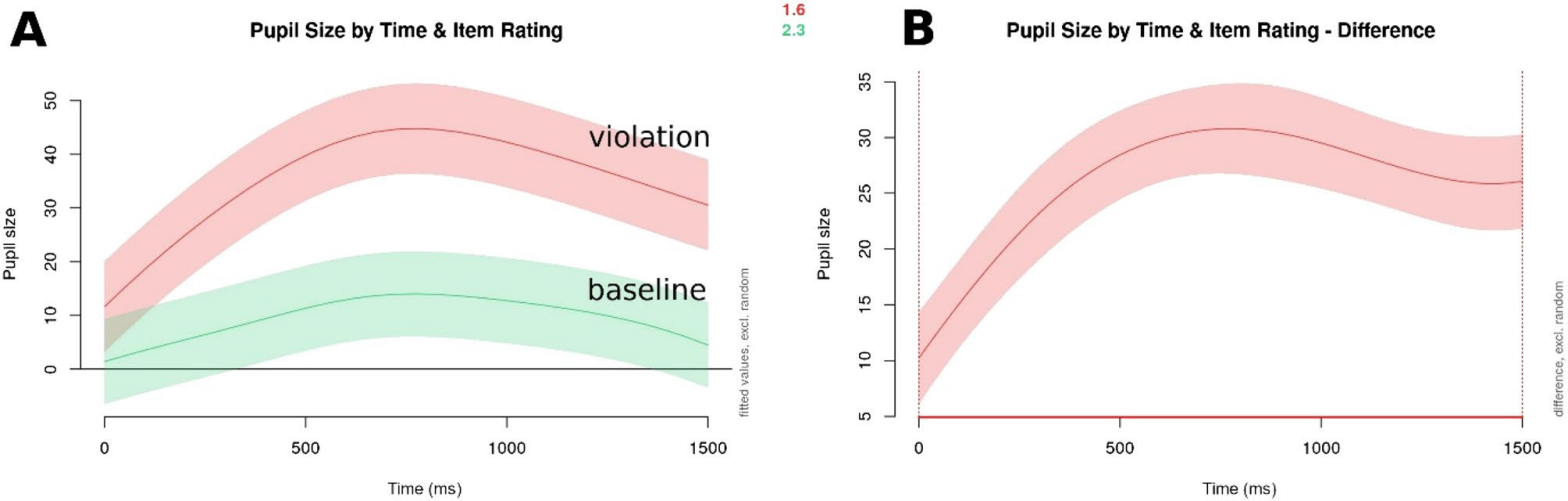
Visualizations of pupil size by item rating over time (in ms since target word onset). Panel A shows baseline (green/lower curve) vs. socio-cultural violation condition (red/upper curve), and Panel B visualizes the difference between the two, with the red line on the x-axis (below the plot) highlighting the time frame where the difference in pupil size between the baseline and error conditions is significantly different. The shaded areas denote a 95% confidence interval.

Just as for morpho-syntactic errors and semantic anomalies, item rating emerged as a significant predictor in an interaction with time since target word onset (see the model summary in Table 9, and Fig. 5; χ^2^ (5) = 408.864, *p* < 0.001 as compared to a basic model containing only time as a predictor variable, with the same random structure).

**Table 9:** Summary output for the basic GAMM modelling participants' pupil sizes in response to socio-cultural violations.

Term	edf	ref.df	Statistic	*p* value	*p* level
s (time)	5.907	6.614	23.14	< 0.001	***
s (rating)	8.915	8.996	157.708	< 0.001	***
ti (time, rating)	11.677	13.595	29.073	< 0.001	***
s (participant, time)	569.411	791	31.874	< 0.001	***
s (item.id)	101.042	102	129.583	< 0.001	***

**Figure 6:**
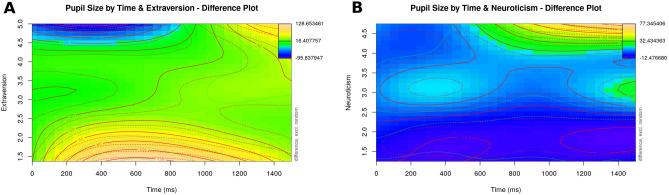
Visualization of the effects of item rating and the listener’s Extraversion (Panel A) and Neuroticism (Panel B) on pupil size over time in response to socio-cultural violations. Plots were generated using the itsadug (Version 2.3^63^) package in R.

In further GAMMs, two traits were found to be significant in interactions with item rating and time:

First, an effect of Extraversion emerged, that is reminiscent of the effect of Extraversion in response to semantic anomalies above: Introverted listeners experienced an early increase in dilation, around 200 ms after target word onset, while highly extraverted listeners showed an increase in pupil size much later, around 1100 ms after target word onset (see the model summary in Table 10, and the visualization in Panel A of Fig. 6; χ^2^ (12) = 418.977, p < 0.001 as compared to a model without the Extraversion variable). Second, the listeners’ Neuroticism also affected processing, where highly neurotic listeners experienced larger changes in pupil size than less neurotic participants starting around 700 ms after the onset of the target word (see the model summary in Table 11, and Panel B of Fig. 6; χ^2^ (12) = 244.861, p < 0.001 as compared to a model without the Neuroticism variable).

**Table 10:** Summary output for the GAMM modelling participants' pupil sizes in response to socio-cultural violations and with their Extraversion score as a predictor.

Term	edf	ref.df	F-statistic	p value	p level
s (time)	5.895	6.599	21.991	< 0.001	***
s (rating)	8.849	8.99	137.756	< 0.001	***
ti (time, rating)	13.177	14.59	29.83	< 0.001	***
s (extr)	1.005	1.006	0.001	0.981	ns
ti (extr, time)	1.028	1.033	0.02	0.893	ns
ti (extr, rating)	15.552	15.936	79.353	< 0.001	***
ti (extr, time, rating)	57.519	61.811	15.506	< 0.001	***
s (participant, time)	570.528	792	32.321	0.004	**

**Table 11:** Summary output for the GAMM modelling participants' pupil sizes in response to socio-cultural violations and with their Neuroticism score as a predictor.

Term	edf	ref.df	F-statistic	p value	p level
s (time)	5.853	6.562	20.246	< 0.001	***
s (rating)	8.901	8.995	134.133	< 0.001	***
ti (time, rating)	13.03	14.46	24.68	< 0.001	***
s (neur)	1.009	1.009	2.272	0.132	ns
ti (neur, time)	2.262	2.401	1.773	0.136	ns
ti (neur, rating)	15.625	15.956	38.026	< 0.001	***
ti (neur, time, rating)	55.639	60.624	15.276	< 0.001	***
s (participant, time)	566.374	792	31.325	< 0.001	***
s (item.id)	101.047	102	130.315	< 0.001	***

## Discussion

We investigated the extent to which listeners’ Big Five traits would predict their processing of spoken sentences with morpho-syntactic, semantic, and socio-cultural violations. The results suggest that the listener’s personality traits modulate the resource allocation or processing load that participants experience during online language comprehension, albeit differently depending on the linguistic phenomenon.

These results are in line with our expectations set out initially, in that all three violation types were associated with a significant increase in pupil size, and that further, these significant differences in pupil dilation were modulated by the participant’s personality. Results are supportive of findings from prior research suggesting that language comprehension is directly and immediately reflective of context, including states of the real-world, and the listener’s experiences within them, adding listeners’ personality among factors affecting moment-by-moment language comprehension^33^. Much like the results reported by Van Berkum and colleagues^7^, our results suggest that both semantic violations and socio-cultural violations elicit an effect in the same time frame, with effects emerging as early as 200-300 ms after the onset of the critical word, suggesting that both types of information are considered concurrently^5–7^.

While our results, observing significantly different pupil dilations in response to three different types of violations, suggest a significant difference in processing load that is modulated by personality traits, we cannot pinpoint the precise origin of these effects; as pupillometry has been shown to be sensitive to effects stemming from increased processing or affective demands^34,36,37^, it may well be that there is an emotional component to our results. This is especially so since personality traits are correlated with individuals' values and world view^12,47,66^, and statements conflicting with an individual's values have been previously shown to rapidly engage the affect system^7^. It is further important to note that no one Big Five trait predicted pupil dilation in response to all three types of violations; instead, the three different types (morpho-syntactic errors, semantic anomalies, and socio-cultural violations) elicited distinct patterns (an overview is presented in Table 12).

**Table 12:** Overview of effects of item rating/condition, and interaction effects with Big Five traits on pupil size, as reported in the Results section; a upwards arrow indicates a larger pupil dilation.

	Morpho-syntactic errors	Semantic anomalies	Socio-cultural violations
Item rating	↑	↑	↑
Big Five × Item rating			
Openness	Less open: ↑	*No effect*	*No effect*
Conscientiousness	*No effect*	*No effect*	*No effect*
Extraversion	*No effect*	Less extraverted: early ↑ More extraverted: late ↑	Less extraverted: early ↑ More extraverted: late ↑
Agreeableness	*No effect*	Less agreeable: ↑	*No effect*
Neuroticism	*No effect*	*No effect*	More neurotic: ↑

The only Big Five trait to affect pupillary responses to morpho-syntactic errors (agreement violations) was Openness, where less open listeners experienced significant pupil dilation when encountering an error. This is in line with findings from Boland and Queen’s^33^ off-line ratings study, and shows that real-time language comprehension is likewise modulated by the Openness trait. Results thus suggest that individuals that are generally less inventive, creative, and unconventional (cf. Table 1) experience a larger processing difficulty after encountering a morpho-syntactic error than their more open peers; as such, a deviation from linguistic norms, even if the resulting sentence can still be easily interpreted semantically and pragmatically, seems to be associated with a higher cognitive processing load for less open individuals.

For semantic anomalies (“People read *heads* in bed”) two personality predictors, Agreeableness and Extraversion, were found to be significant. Firstly, less agreeable listeners, individuals that would be described as less cooperative, trustful, and sympathetic (cf. Table 1), experienced significantly larger pupil dilation than their more agreeable peers when they encountered a semantic anomaly. This is in line with findings in Boland and Queen^33^, where “grammos,” such as *to* for *too* or *it’s* for *its*, resulted in less agreeable readers rating housemates as significantly worse than their more agreeable peers. Based on these results, the authors describe less agreeable individuals as generally “less tolerant of deviations from convention” (p. 10), an interpretation supported by our results.

Secondly, less extraverted listeners were found to experience significant pupil dilation rather early (around 200 ms) after the onset of the semantic anomaly. However, an additional significant increase in pupil size was observed later, around 800–1,000 ms after the semantic anomaly, for highly extraverted listeners. We will return to this effect below.

In the socio-cultural violation condition, where, for example, a male speaker would produce an utterance like “I always buy my bras at Hudson’s Bay”, two Big Five traits emerged as significant predictors in interactions with time since target word onset and item rating. More neurotic individuals experienced a significantly larger change in pupil size than their less neurotic peers starting around 700 ms after target word onset. This is an intuitively accessible effect, since individuals high on the Neuroticism scale are associated with attributes such as sensitivity, nervousness, and irritability—they generally experience greater difficulty dealing with novel or unexpected stimuli. Interestingly, no such effect of Neuroticism was found in the processing of semantic anomalies; we suspect that this may have to do with the difference between purely semantic anomalies and socio-cultural violations, which involve the processing of stereotypes, discussed in greater detail further below, and are closely intertwined with social identity; it seems that the listener’s Neuroticism modulates the processing of social identity-related violations, but not that of purely semantic anomalies.

The second significant personality effect for socio-cultural violations was Extraversion, where more introverted listeners experienced a significant pupil dilation early (around 200 ms), and extraverted listeners experienced the same in a much later time frame (around 1100 ms). Note that this effect is very similar to the effect of Extraversion on the processing of semantic anomalies (compare Figs. 4, 5, 6), albeit stronger.

Extraversion was the only Big Five trait that emerged as a significant predictor for more than one type of violation. For both semantic anomalies and socio-cultural violations, it was introverted listeners who experienced an early dilation, and extraverted listeners who experienced a late dilation. We did not observe a polar effect pattern like this one for any other Big Five trait in our study, suggesting that the Extraversion trait may have special significance for the comprehension of violations that rely on linguistic or social meaning rather than purely linguistic form. The pattern of effects suggests that introverted listeners either experience surprisal or difficulty with lexical integration at the time of the violation, resulting in significant allocation of resources to the violation, very early on, whereas extraverted listeners’ processing seems to be affected by the violation much later. Comparing our pupillometric results to the findings from a behavioural ratings experiment, while the clashing sentences in all three clash types were rated significantly less acceptable than the correct/non-clashing sentences, it is interesting to note that the rating of socio-cultural clashes was not significantly associated with differences in the listener’s Extraversion score (however, the ratings of morpho-syntactic errors and semantic anomalies were)^47^. This may be due to task differences: namely due to the coarser, offline nature of the acceptability ratings paradigm, which cannot capture fleeting, time-sensitive physiological responses.

Of the Big Five traits, Extraversion is the one most closely related to how an individual interacts with others; extraverts are described as outgoing and energetic, as enjoying large gatherings, and generally enjoying socialization. It is the most “social” trait among the Big Five, and as such, may have a special place regarding language comprehension. For example, in Boland and Queen^33^, Extraversion interacted with the presence of “typos” and “grammos”: more extraverted people were more likely to overlook errors, whereas introverts would judge authors of error-specked emails more harshly as a potential housemate. The importance of Extraversion as a highly social trait is further supported by research suggesting it is the number one trait that mothers view as desirable in their children^67^, and by findings from face-recognition research: Extraversion modulates amygdala activation when viewing happy faces^68^; correlates with greater aptitude at recognizing faces, independently of general cognitive or object-recognition skill^69^; and modulates the use of gaze cues in interaction with facial emotions^70^.

The Introversion/Extraversion scale, as one of the three dimensions in Eysenck’s theory of personality, has had a long standing in psychological research^25^. Eysenck’s theory assumes that all individuals attempt to operate at optimal levels of arousal in contrast to introverts who generally operate at an optimal (or already heightened) level of arousal and need not seek out additional stimulation, extraverts are assumed to operate at sub-optimal arousal levels and thus crave additional stimulation^24^. Even though the pupillometry paradigm is not particularly well-suited to assess the fine-grained timing of a process, our results suggest that introverts experienced difficulty immediately at the target word^50,51^ In the context of Eysenck’s personality theory, our results suggest that, during the initial processing of semantic anomalies and socio-cultural violations, introverted participants may have experienced heightened arousal compared to their more extroverted peers, reflected in the significantly larger early pupil dilation. The late effect we observed for more extraverted participants may suggest difficulty at the integration/situation model stage, that is, while integrating the information given in the sentence into a coherent representation^71,72^.

Importantly, Extraversion relates to both how much an individual interacts with people generally and to how much someone interacts with people that are not like themselves, and that do not speak like they do. Through modulating exposure to social interaction, and specifically to more diverse social interaction, Extraversion thus has the potential to modulate how often someone experiences non-canonical, (subjectively) unusual stimuli, or stimuli that, more generally speaking, do not match the listener’s prior experience or their world view. Considering this pervasiveness, it may be that the trait modulates prior exposure to similar (non-canonical) stimuli, and, through this, the comprehension of a violation. This highlights three things: Firstly, the crucial role of the individual’s prior experience in language comprehension; secondly, the intertwined nature of an individual’s personality with other aspects of their life, such as exposure to varied socio-cultural settings and utterances; and lastly, the difference between agreement violations as actual linguistic errors on the one hand, and semantic anomalies and stereotype-related violations—not errors in the same, normative sense, but rather “weird” statements that are unexpected, but could be acceptable in an imaginary world, if placed into a suitable context^73^—on the other.

A crucial manipulation in our study involved statements that are either congruous or incongruous with established gender stereotypes. Stereotypes are “cognitive shortcuts” that facilitate stimulus processing in a complex world. They make it possible for individuals to rapidly categorize other people based on a few salient characteristics, without actually getting to know the person in detail. This individual is then expected to behave like a member of the category in question^74^. Gender stereotypes are considered to be part of world knowledge, and to be activated immediately in language comprehension^75^. This immediate activation seems to be largely automatic, and difficult to suppress^76^; and it also seems to occur even when gender stereotypes are not required for comprehension, or for establishing coherence^75,77^. Social knowledge based on gender stereotypes even has the potential to override syntactic information^78^.

Prior literature has found consistent links between two Big Five traits, namely low Openness and low Agreeableness, and prejudicial tendencies^79,80^. However, no link has been established between those two traits and the linguistic processing of prejudicial statements. We expected listeners with low Agreeableness or low Openness to experience larger pupil dilation when encountering a socio-cultural violation, which relies on gender stereotyping and thus may elicit a stronger response from less agreeable and less open individuals. Interestingly, however, neither emerged as a significant predictor for pupillary responses to this type of violation. This suggests that prior experience with, and exposure to, novel or less common statements or situations, may exert more influence on the processing of statements relating to established stereotypes than an abstract personality trait, such as Conscientiousness, by itself. These close ties between an individual’s personality and their lived experience on the one hand, and between these two facets and resource allocation during language comprehension on the other^11,17^, suggest that an individual’s personality, their experience in the real world, and how they comprehend language are inextricably linked.

While our study was not designed to discriminate between different sentence processing or language comprehension theories, and we did not assess our participants' WMC, our results suggest that listeners, based on individual differences in personality traits, experience different levels of processing effort when encountering different types of violations. Our results are thus broadly in line with constraint-based models of sentence processing^7,81–83^, and theories of syntactic parsing that consider extra-linguistic information at an early stage^84–87^. Future research may want to investigate the role of WMC in regards to the processing of different types of violations, which may provide important insight into the role of WMC in regards to semantic expectations and the deployment of cognitive resources, especially in interaction with personality traits^8^.

To sum up, our findings suggest that personality traits modulate the resource allocation that a listener experiences when processing sentences that violate expectation in different ways. These results add to the body of research on the influence of individual differences and extra-linguistic information on the immediate, incremental processing of language. Our results are in line with a view of language processing that considers multiple sources of information, including speaker and listener related individual differences, in parallel, suggesting that language processing interacts with aspects of general cognition from the earliest moments. Our study is the first to show that individuals with different personality profiles exhibit different patterns of resource allocation during real-time language comprehension.

## Supplementary Information

Supplementary Information 1.

Supplementary Information 2.

## Data Availability

The datasets generated during and/or analysed during the current study are available from the corresponding author on reasonable request.
