# Supplementary material for: Listener’s personality traits predict changes in pupil size during auditory language comprehension
Source: Sci Rep. 2021 Mar 8;11:5443. doi: 10.1038/s41598-021-84886-3 (PMC7940482; doi:10.1038/s41598-021-84886-3)
Supplement: Supplementary file 1 — Supplementary Information 1. [file 41598_2021_84886_MOESM1_ESM.docx]

**SUPPLEMENTARY MATERIALS**

**Experiment Stimuli – Anomalous Statements**

**Morpho-Syntactic Errors**

He frequently walk his dog in the park downtown.

She seldom swim three miles at the pool nearby.

You always speaks the truth when I ask you to.

We often sings old songs together at camp.

They normally eats their lunch inside the building.

I seldom buys things for myself or my friends.

He always ride his bike to work in June.

They often buys their milk at the store down the street.

She usually drive her car slowly in the snow.

They rarely wraps any gifts for a friend’s birthday.

She always give her son money for candy.

They often helps their friends with their homework.

I often reads a book on long-haul flights.

He often speak Turkish during recess.

We never cooks dinner at home on Fridays.

We seldom sends postcards from our vacation.

He usually eat bacon in the morning.

She never take the bus to the library.

He constantly drive his car around the city.

She normally fly to Europe around Christmas.

He frequently have burgers for dinner after work

She always watch shows on her friend’s Netflix.

I constantly wears my watch, even at night.

You rarely comes over to my house anymore.

We frequently travels places in the summer.

She sometimes read books on arts and crafts.

He sometimes listen to songs from his childhood.

She usually ride transit to get to school.

**Semantic Anomalies**

Bees often collect storage in our backyard.

Cats frequently hunt bricks around their homes.

Students seldom forget dancers in the locker room.

Dancers usually wear limbs at a performance.

Grandma always cleans the cheek by herself.

We always hear skies in the forest.

He never catches firms thrown with a spin.

She rarely attends use in the morning.

People often read heads for pleasure at night.

She sometimes waters terms around the block.

Bikers constantly fear scales in their tires.

Dogs sometimes chase teas on the road for fun.

Bosses normally pay their cups weekly or monthly.

Her mum frequently writes cultures to friends in Europe.

Plants usually need money to grow and bloom.

Lisa rarely cooks chart with rice for dinner.

**Socio-Cultural Violations**

***Produced by male speaker:***

I usually wear lip gloss to work and at home.

I always enjoy knitting in my free time.

I normally wear high heels to formal parties.

I constantly help my mom with cooking and chores.

I usually fix the holes in my clothes myself.

I sometimes buy my bras at Hudson’s Bay.

I normally shave my legs every three days.

I usually avoid tampons as I prefer pads.

I frequently apply perfume in the mornings.

I often drink wine on the weekends.

I often prepare muffins for block parties.

I always enjoy coloring when I am stressed.

I sometimes fight the good fight for the homeless.

I always watch movies on fashion with friends.

I frequently buy new soap for the kitchen.

I constantly wear dresses to board meetings.

I constantly carry a purse with all my things.

I normally wipe the table after we had lunch.

I frequently play Wii games with my friends.

I sometimes grow my hair quite long for fun.

I usually prefer reading over coloring.

I often visit my friends when I feel lonely.

I usually go to the spa to relax after work.

I often wear flowers in my hair for work.

I always wear hair bands to hold in my bangs.

I frequently read gossip news during lunch break.

I often work with kids in my daytime job.

I normally clean the floor with a soft sponge.

I constantly think of home when I travel.

I sometimes shave my arms in the summer months.

***Produced by female speaker:***

I usually wear blue jeans to work and at home.

I always enjoy football in my free time.

I normally wear dress shoes to formal parties.

I constantly help my dad with garden work.

I usually fix the brakes on my Dodge myself.

I sometimes buy my ties at Hudson’s Bay.

I normally shave my beard every three days.

I usually avoid urinals as I prefer stalls.

I frequently apply cologne in the mornings.

I often drink beer on the weekends.

I often prepare spare ribs for block parties.

I always enjoy gaming when I am stressed.

I sometimes fight my brothers over stupid stuff.

I always watch movies about war with my friends.

I frequently buy new coal for the barbecue.

I constantly wear suits to board meetings.

I constantly carry a gun to defend myself.

I normally wipe my hard drive every few months.

I frequently play war games with my buddies.

I sometimes grow my beard quite long for fun.

I usually prefer hockey over football.

I often visit strip clubs when I feel lonely.

I usually go to the bar to relax after work.

I often wear cufflinks on my shirts for work.

I always wear a hat to cover my head.

I frequently read sports news during lunch break.

I often work on cars in my daytime job.

I normally clean the car with a soft sponge.

I constantly think of sex when I am home.

I sometimes shave my chest in the summer months.

**Non-Anomalous Baseline Items**

**Not Dependent on Speaker Gender:**

He frequently walks his dog in the park downtown.

She seldom swims three miles at the pool nearby.

They often buy their milk at the store down the street.

You always speak the truth when I ask you to.

We often sing old songs together at camp.

They normally eat their lunch inside the building.

I seldom buy things for myself or my friends.

He always rides his bike to work in June.

She usually drives her car slowly in the snow.

They rarely wrap any gifts for a friend’s birthday.

She always gives her son money for candy.

They often help their friends with their homework.

I often read a book on long-haul flights.

He often speaks Turkish during recess.

We never cook dinner at home on Fridays.

We seldom send postcards from our vacation.

He usually eats bacon in the morning.

She never takes the bus to the library.

He constantly drives his car around the city.

She normally flies to Europe around Christmas.

He frequently has burgers for dinner after work.

She always watches shows on her friend’s Netflix.

I constantly wear my watch, even at night.

You rarely come over to my house anymore.

We frequently travel places in the summer.

She sometimes reads books on arts and crafts.

He sometimes listens to songs from his childhood.

She usually rides transit to get to school.

Cats frequently hunt mice around their homes.

Students seldom forget towels in the locker room.

Dancers usually wear skirts at a performance.

Grandma always cleans the house by herself.

We always hear birds in the forest.

He never catches balls thrown with a spin.

She rarely attends class in the morning.

People often read books for pleasure at night.

She sometimes waters plants around the block.

Bikers constantly fear flats on their tires.

Dogs sometimes chase bikes on the road for fun.

Bosses normally pay their staff weekly or monthly.

Her mum frequently writes letters to friends in Europe.

Plants usually need water to grow and bloom.

Lisa rarely cooks fish with rice for dinner.

Cats often catch mice in the night.

Dogs generally fetch sticks on a walk.

Rabbits often change colour in the winter.

Seagulls often catch fish in groups.

Horses generally accept being trained.

Birds often sing loudly in the morning.

Foxes usually live alone in the forest.

Wolves seldom enjoy being near humans.

Chickens normally live in a coop.

Roosters often announce the sunrise.

Bears sometimes approach picknickers.

Moose sometimes block the roads in a park.

Geese always watch out for their young.

Goslings generally follow their mother.

Ants usually build large anthills.

Bees generally make honey from pollen.

Bugs often live where it is humid.

Skunks often spray their enemies.

Donkeys often carry heavy loads.

Rabbits generally enjoy eating carrots.

Dogs typically bark at intruders.

Cats often purr when they are content.

Birds frequently sit perched atop trees.

Chickens generally lay many eggs.

Hippos never jump high into the air.

Giraffes always have very long necks.

Rhinos usually have large horns.

Beavers often build dams in rivers.

Monkeys generally enjoy bananas.

Lions frequently roar very loudly.

Leopards generally run very fast.

Bears seldom venture into cities.

**Dependent on Speaker Gender**

***Produced by male speaker:***

I usually wear blue jeans to work and at home.

I always enjoy football in my free time.

I normally wear dress shoes to formal parties.

I constantly help my dad with garden work.

I usually fix the brakes on my Dodge myself.

I sometimes buy my ties at Hudson’s Bay.

I normally shave my beard every three days.

I usually avoid urinals as I prefer stalls.

I frequently apply cologne in the mornings.

I often drink beer on the weekends.

I often prepare spare ribs for block parties.

I always enjoy gaming when I am stressed.

I sometimes fight my brothers over stupid stuff.

I always watch movies about war with my friends.

I frequently buy new coal for the barbecue.

I constantly wear suits to board meetings.

I constantly carry a gun to defend myself.

I normally wipe my hard drive every few months.

I frequently play war games with my buddies.

I sometimes grow my beard quite long for fun.

I usually prefer hockey over football.

I often visit strip clubs when I feel lonely.

I usually go to the bar to relax after work.

I often wear cufflinks on my shirts for work.

I always wear a hat to cover my head.

I frequently read sports news during lunch break.

I often work on cars in my daytime job.

I normally clean the car with a soft sponge.

I constantly think of sex when I am home.

I sometimes shave my chest in the summer months.

***Produced by female speaker:***

I usually wear lip gloss to work and at home.

I always enjoy knitting in my free time.

I normally wear high heels to formal parties.

I constantly help my mom with cooking and chores.

I usually fix the holes in my clothes myself.

I sometimes buy my bras at Hudson’s Bay.

I normally shave my legs every three days.

I usually avoid tampons as I prefer pads.

I frequently apply perfume in the mornings.

I often drink wine on the weekends.

I often prepare muffins for block parties.

I always enjoy coloring when I am stressed.

I sometimes fight the good fight for the homeless.

I always watch movies on fashion with friends.

I frequently buy new soap for the kitchen.

I constantly wear dresses to board meetings.

I constantly carry a purse with all my things.

I normally wipe the table after we had lunch.

I frequently play Wii games with my friends.

I sometimes grow my hair quite long for fun.

I usually prefer reading over coloring.

I often visit my friends when I feel lonely.

I usually go to the spa to relax after work.

I often wear flowers in my hair for work.

I always wear hair bands to hold in my bangs.

I frequently read gossip news during lunch break.

I often work with kids in my daytime job.

I normally clean the floor with a soft sponge.

I constantly think of home when I travel.

I sometimes shave my arms in the summer months.
