# Supplementary material for: Listener’s personality traits predict changes in pupil size during auditory language comprehension
Source: Sci Rep. 2021 Mar 8;11:5443. doi: 10.1038/s41598-021-84886-3 (PMC7940482; doi:10.1038/s41598-021-84886-3)
Supplement: Supplementary file 2 — Supplementary Information 2. [file 41598_2021_84886_MOESM2_ESM.docx]

# Supplementary Information

Listener’s Personality Traits Predict Changes in Pupil Size During Auditory Language Comprehension

Isabell Hubert Lyall^1,*^ and Juhani Järvikivi^1^

^1^ Department of Linguistics, University of Alberta, Edmonton, AB, T6G 2R3, Canada

The distributions of Extraversion, Neuroticism, and Agreeableness scores differed significantly between male and female participants: In two-tailed t-tests, male participants were found to be significantly less extraverted (mean[SD]*_male_* = 2.79 [0.81], mean[SD]*_female_* = 3.33 [0.91]; t (26.41) = 2.39, p = 0.02), less neurotic (mean[SD]*_male_* = 2.74 [0.81], mean[SD]*_female_* = 3.40 [0.74]; t (22.84) = 3.04, p = 0.006), and less agreeable (mean[SD]*_male_* = 3.56 [0.70], mean[SD]*_female_* = 3.95 [0.56]; t (21.15) = 2.14, p = 0.04) than female participants, although the latter difference was only found to be marginally significant. The two gender groups did not differ significantly in Openness or Conscientiousness scores (p = 0.53 and 0.46, respectively). Similarly, in a two-tailed t-test, externally recruited participants were found to be significantly less neurotic (mean[SD]*_external_* = 2.96 [0.70], mean[SD]*_internal_* = 3.38 [0.80]; t (41.19) = 2.33, p = 0.02) than internally recruited participants. See Table 1 and Table 2, and Figure 1 and Figure 2 in this Supplementary Information document for details.

We are providing information on the mean and spread of the personality traits observed in our participant sample in the context of values in the literature in Table 3.

A two-tailed t-test revealed that the age distribution differed significantly between the two recruitment strategies (mean [SD]external = 33.5 [19.7], mean [SD]internal = 19.6 [2.3]; t (21.19) = -3.30, p = 0.003). A cross-tabulation of age by recruitment strategy can be found in Table 4, with a visualization presented in Figure 3.

| ***Trait*** | | **Min / Max** | **Mean [SD]** |
| --- | --- | --- | --- |
| **Openness** | | 1.9 / 4.8 | 3.4 [0.7] |
|  | female | 1.9 / 4.8 | 3.4 [0.7] |
|  | male | 2.0 / 4.6 | 3.3 [0.7] |
| **Conscientiousness** | | 2.2 / 4.9 | 3.6 [0.6] |
|  | female | 2.4 / 4.9 | 3.6 [0.6] |
|  | male | 2.2 / 4.3 | 3.5 [0.6] |
| **Extraversion** | | 1.4 / 5.0 | 3.2 [0.9] |
|  | female | 1.4 / 5.0 | 3.3 [0.9] |
|  | male | 1.8 / 4.5 | 2.8 [0.8] |
| **Agreeableness** | | 1.8 / 4.9 | 3.9 [0.6] |
|  | female | 2.6 / 4.9 | 4.0 [0.6] |
|  | male | 1.7 / 4.7 | 3.6 [0.7] |
| **Neuroticism** | | 1.3 / 4.8 | 3.3 [0.8] |
|  | female | 1.6 / 4.8 | 3.4 [0.7] |
|  | male | 1.3 / 4.5 | 2.7 [0.8] |

Table 1: Distributions of Big Five traits by participant gender; n_female_ = 71, n_male_ = 17.

| ***Trait*** | | **Min / Max** | **Mean [SD]** |
| --- | --- | --- | --- |
| **Openness** | | 1.9 / 4.8 | 3.4 [0.7] |
|  | internal | 1.9 / 4.8 | 3.3 [0.7] |
|  | external | 2.3 / 4.7 | 3.6 [0.7] |
| **Conscientiousness** | | 2.2 / 4.9 | 3.6 [0.6] |
|  | internal | 2.2 / 4.9 | 3.5 [0.6] |
|  | external | 2.7 / 4.3 | 3.6 [0.5] |
| **Extraversion** | | 1.4 / 5.0 | 3.2 [0.9] |
|  | internal | 1.4 / 5.0 | 3.3 [0.9] |
|  | external | 1.4 / 4.4 | 3.0 [0.9] |
| **Agreeableness** | | 1.8 / 4.9 | 3.9 [0.6] |
|  | internal | 1.8 / 4.9 | 3.9 [0.6] |
|  | external | 2.6 / 4.8 | 3.8 [0.5] |
| **Neuroticism** | | 1.3 / 4.8 | 3.3 [0.8] |
|  | internal | 1.3 / 4.8 | 3.4 [0.8] |
|  | external | 1.6 / 4.1 | 3.0 [0.7] |

Table 2: Distributions of Big Five traits by recruitment strategy; n_internal_ = 66, n_external_ = 22.


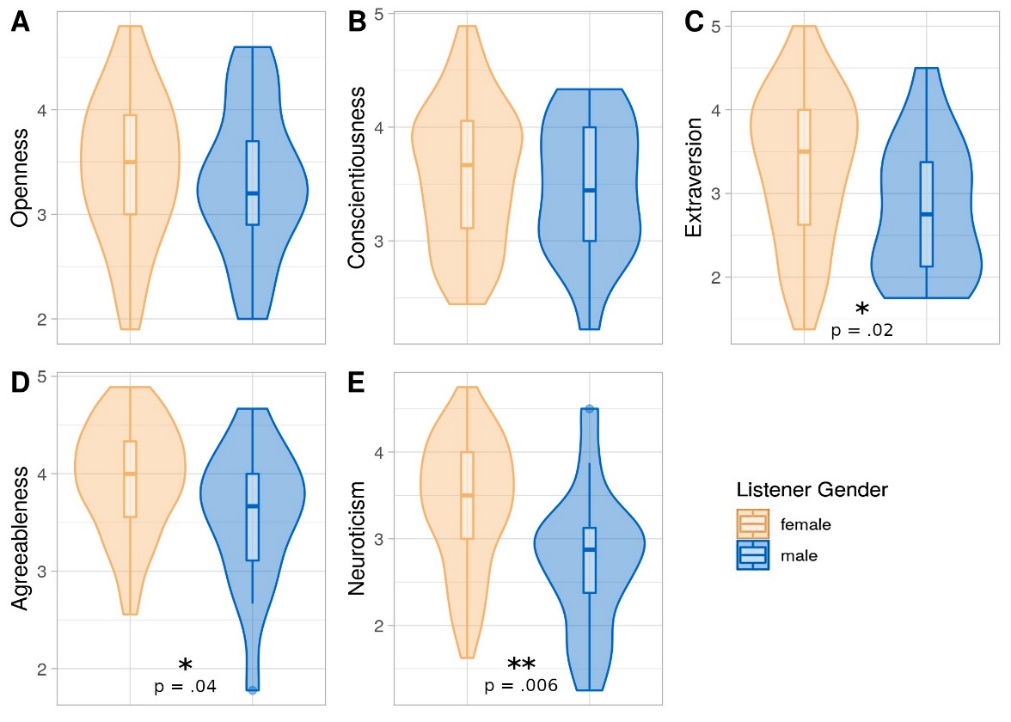


Figure 1: Personality trait score distribution by listener gender. Plot was generated using the ggplot2 (Version 3.2.1^64^) and ggpubr (Version 0.3.0^65^) packages in R.


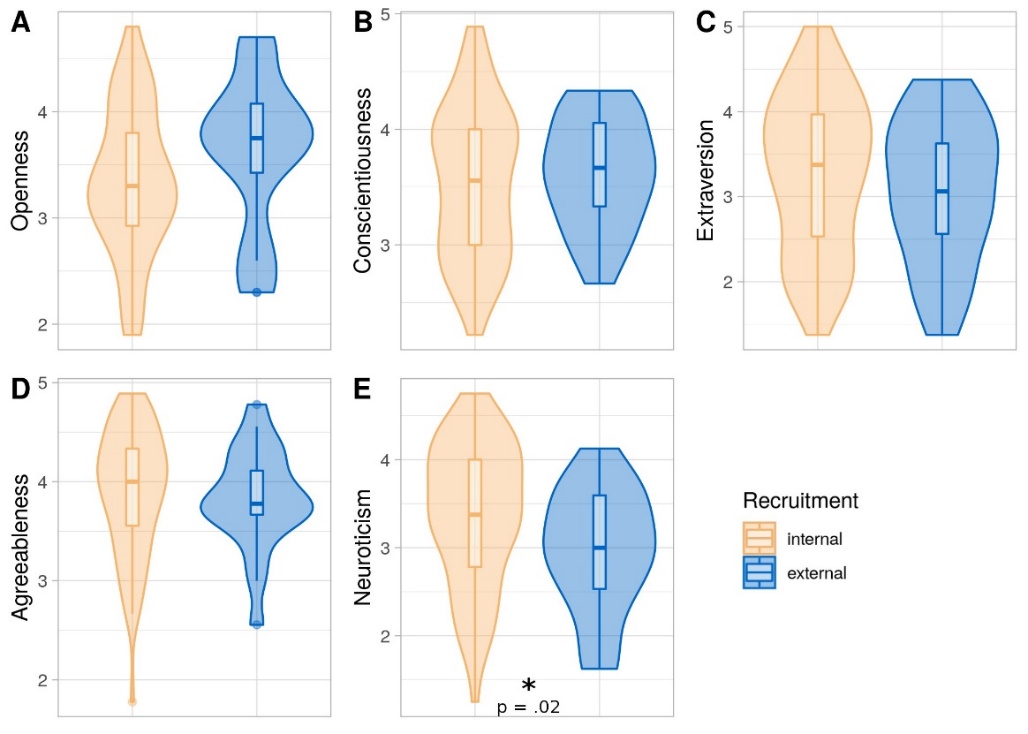


Figure 2: Personality trait distribution by recruitment. Plot was generated using the ggplot2 (Version 3.2.1^64^) and ggpubr (Version 0.3.0^65^) packages in R.

|  |  | **Openness** | | **Consc.** | | **Extraversion** | | **Agreeableness** | | **Neuroticism** | |
| --- | --- | --- | --- | --- | --- | --- | --- | --- | --- | --- | --- |
| *Source* | *n =* | Mean | SD | Mean | SD | Mean | SD | Mean | SD | Mean | SD |
| **Boland & Queen (2016)** | 83 | 3.68 | 0.64 | 3.92 | 0.71 | 3.05 | 0.77 | 3.69 | 0.62 | 2.48 | 0.87 |
| **Gurven et al. (2013)** | 632 | 3.01 | 0.46 | 3.03 | 0.5 | 2.53 | 0.48 | 3.41 | 0.44 | 2.44 | 0.39 |
| **ISDP (2008)** *men* | 5,445 | 3.71 | 0.60 | 3.39 | 0.66 | 3.32 | 0.68 | 3.57 | 0.59 | 2.79 | 0.72 |
| **ISDP (2008)** *women* | 7,798 | 3.68 | 0.60 | 3.5 | 0.66 | 3.43 | 0.73 | 3.68 | 0.60 | 3.14 | 0.75 |
| **Rammstedt (2007)** | 2,569 | 3.41 | 0.88 | 4.10 | 0.69 | 3.24 | 0.88 | 3.2 | 0.83 | 3.49 | 0.85 |
| **Srivastava et al. (2003)** age = 23 | 4,828 | 3.94 | 0.66 | 3.52 | 0.70 | 3.30 | 0.89 | 3.64 | 0.70 | 3.28 | 0.82 |
| **This paper** | 88 | 3.42 | 0.70 | 3.56 | 0.61 | 3.22 | 0.91 | 3.88 | 0.60 | 3.27 | 0.80 |

Table 3: Comparison between Big Five trait distributions observed in the literature and in the participant sample for this study; ISDP data is from Schmitt & Shackelford (2008).

|  | *Age* |  |
| --- | --- | --- |
| *Recruitment* | **Min / Max** | **Mean [SD]** |
| **Internal** | 17 / 30 | 19.6 [2.3] |
| **External** | 19 / 83 | 33.5 [19.7] |

Table 4: Cross-tabulation of participant age by recruitment strategy.


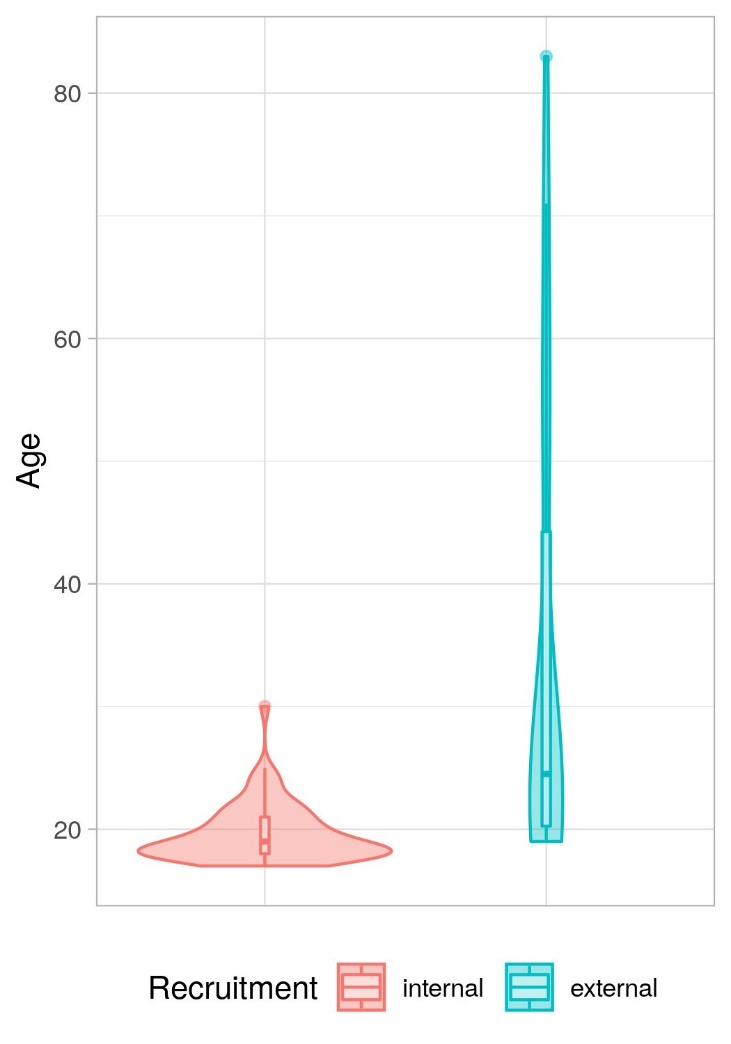


Figure 3: Visualization of participant age by recruitment strategy. Plot was generated using the ggplot2 (Version 3.2.1^64^) package in R.
